# Exercise capacity in moderate aortic stenosis: a cardiopulmonary stress echocardiography study

**DOI:** 10.1186/s44156-025-00070-7

**Published:** 2025-03-05

**Authors:** Sveeta Badiani, Jet van Zalen, Sahar Alborikan, Aeshah Althunayyan, David Bruce, Thomas Treibel, Sanjeev Bhattacharyya, Nikhil Patel, Guy Lloyd

**Affiliations:** 1 Barts Heart Centre, St Bartholomew’s Hospital, Barts Health NHS Trust, London, UK; 2 William Harvey Research Institute, Queen Mary University of London, London, UK; 3 Eastbourne District General Hospital, Kings Drive, Eastbourne, UK; 4 Maidstone and Tunbridge Wells NHS Trust, Maidstone, Kent, UK; 5 Cardiac Centre, King Fahad Specialist Hospital, Damman, Saudi Arabia

**Keywords:** Aortic stenosis, Stress echocardiography, Cardiopulmonary exercise testing

## Abstract

**Background:**

Patients with moderate aortic stenosis (AS) may experience symptoms and adverse outcomes. The aim of this study was to determine whether patients with moderate AS exhibited objective evidence of exercise limitation, compared with age and sex matched controls and if so, to determine which echocardiographic parameters predicted exercise ability.

**Methods:**

This was a prospective case control study of patients with moderate AS (peak velocity (Vmax) 3.0–3.9 m/s, mean gradient (MG) 20-39mmHg, aortic valve area (AVA)1.1-1.5cm^2^ ) and left ventricular ejection fraction (LVEF) ≥ 55%. All patients underwent cardiopulmonary stress echocardiography.

**Results:**

25 patients with moderate AS (Vmax 3.5 ± 0.2mmHg, mean gradient 28 ± 5mmHg, AVA 1.2 ± 0.1cm^2^, LVEF 61 ± 4%) were compared with 25 controls. % predicted oxygen uptake efficiency slope (OUES), % predicted O_2_ pulse and VO_2_ at anaerobic threshold (AT) were significantly lower in patients compared with controls (OUES 79 ± 15 vs. 89 ± 15%, *p* = 0.013). VO_2_ did not significantly differ between cases and controls.

**Conclusion:**

Objective measures of exercise capacity including OUES, O_2_ pulse and VO_2_ at AT are significantly lower in patients with moderate AS compared with controls, suggesting that these parameters may be more useful than VO_2_ where patients may be unable to complete a maximal exercise test. Risk stratification using cardiopulmonary exercise echocardiography may help to identify patients with moderate AS who are at increased risk of cardiovascular events and should be considered for more intensive surveillance and intervention.

**Trial registration:**

Clinical trial number MRC 0225 IRAS 207395.

**Supplementary Information:**

The online version contains supplementary material available at 10.1186/s44156-025-00070-7.

## Introduction

Aortic valve replacement (AVR) may be considered in patients with moderate aortic stenosis (AS) who are undergoing surgery for other indications. The ACC/AHA guidelines recommend echocardiography surveillance in patients with moderate AS every one to two years, whereas the ESC/EACTS guidelines recommend yearly screening [1–2]. The decision to recommend non-operative treatment in moderate AS has historically been based on (1) the observation of a low overall risk for sudden cardiac death and (2) a high procedural risk associated with AVR [3].

Earlier studies of patients with moderate AS suggested a relatively benign prognosis [4–5]; however later studies suggest that both progression to severe AS occurs at a very high rate and that the clinical outcomes are unfavourable [6–7]. There is growing evidence that the guideline based treatment paradigm may lead to intervention after significant cardiac damage has occurred, with resultant poorer outcomes. A large study in patients with moderate AS found that despite adjustment for LV dysfunction, patients had a 5-year mortality of 56% which was similar to the 67% mortality of patients with severe AS [8].

Some patients with symptoms of decompensation may only have moderate AS yet have no other pathologies to which their symptoms can be attributed. The distinction between asymptomatic patients with severe AS and symptomatic patients with non-severe AS is important [9]. Reduced oxygen consumption on cardiopulmonary exercise testing (CPET) reflects an inability of the heart to deliver oxygen to the periphery, is related to prognosis and assists in the clarification of the aetiology of dyspnoea [10]. This has been shown to be safe and can be combined with stress echocardiography, in the assessment of AS. The aim of this study was to explore whether patients with moderate AS exhibited exercise limitation due to AS or other comorbid factors. We compared exercise performance in patients with moderate aortic AS, with age and sex matched controls, and assessed whether these measures were associated with myocardial recruitment and obstructivity of the valve.

## Methods

This was a prospective, parallel group cohort study of patients with moderate AS. The study complied with the Declaration of Helsinki and relevant ethical and site approvals were obtained. All patients gave written informed consent. Recruitment took place from the valve surveillance clinic at St Bartholomew’s Hospital.

Patients were approached if they were > 18 years of age, had moderate AS on echocardiography (as defined by Vmax 3–3.9 m/s, MG 20-39mmHg, AVA 1.1-1.5cm^2^) and LVEF ≥ 55%. Patients were asked specifically about AS related symptoms and NYHA functional class was documented. Age and gender matched controls were approached to avoid confounding.

Exclusion criteria were co-existing moderate or severe mitral or aortic regurgitation, known chronic lung disease, ischaemic heart disease with evidence of ongoing ischaemia (regional wall motion abnormalities on exercise), inability to cycle, unrecordable echo windows and chronic renal failure with EGFR < 30 ml/min.

### Cardiopulmonary stress echocardiography

Patients underwent a maximal cardiopulmonary exercise test using a ramp protocol, on a semi-recumbent cycle ergometer (ERG 911 S/L, Schiller, Switzerland). Resting respiratory gas exchange and haemodynamic measurements were obtained in the first minute, followed by a 3-minute warm up period. Based on operator assessed functional status, the exercise protocols were determined individually. The work rate (5, 10 or 15 watts) was increased every minute until voluntary exhaustion, aiming for an exercise duration of 8–12 min.

Blood pressure and heart rate were monitored throughout the test. Oxygen uptake, carbon dioxide consumption and ventilation were continuously measured and derived using a calibrated breath-by-breath analyser and averaged every 10 s (Power Cube, Schiller, Switzerland or Quark, Cosmed, Italy). A respiratory exchange ratio (RER > 1.1) indicated a good patient effort. All tests were performed according to the exercise ACC/AHA testing guidelines and tests were terminated if any of the testing guidelines criteria were encountered [11–12].

Peak oxygen uptake (VO_2_ peak) was defined as the highest value recorded during the last 30 s of the exercise test. The ventilatory threshold was determined using the standard triple method and the VE/VCO_2_ slope was calculated up to the respiratory compensation point. A predicted VO_2_ of less than 84% was considered to be reduced [13].

At the end of the exercise test, patients were asked about their symptoms according to the Modified Borg Dyspnoea Scale [14].

Transthoracic echocardiography was performed using GE Vivid E9 or E95 systems (GE Healthcare, Wauwatosa, Wisconsin) with a 4-MHz transducer. All measurements were made as per the British Society of Echocardiography recommendations [15]. Left ventricular ejection fraction (LVEF) was calculated using the Simpson’s biplane method in the apical four-chamber and two-chamber views. Trans-aortic peak and mean gradients were estimated using the modified Bernoulli equation, and the aortic valve area using the continuity equation. Care was taken in assessing the stroke volume index; patients with low flow, low gradient aortic stenosis were excluded.

2D Doppler echocardiography was made at baseline, at low intensity (approximate heart rate 90-100 bpm) and at peak exercise (RER > 1), with continuous live imaging. The echocardiographic dataset encompassed: Apical 4 chamber view with tissue doppler imaging (TDI), aortic valve continuous wave (CW), aortic valve pulsed wave (PW), tricuspid regurgitation peak velocity (TRVmax), apical 4, 2 and 3 chamber views, followed by parasternal long axis (PLAX) and short axis (SAX) views at the papillary muscle level. Pulsed wave tissue doppler imaging (TDI) was used to measure longitudinal systolic myocardial velocities (S’) from the apical 4-chamber view, where the sample volume was placed at the septal and mitral part of the mitral annulus.

Images were optimized for speckle tracking analysis to ensure coverage of the entire ventricle with a frame rate of 50–100 Hz at rest and 70–100 Hz during exercise. Spectral tissue doppler traces was acquired at maximum speed and minimum scale, with an increase in scale on exertion. Global longitudinal strain (GLS) was obtained from the apical views (apical four, three and two chamber).

Images were obtained in real time and digitally stored. Analysis was performed retrospectively and offline (SB), using EchoPAC version 204 (GE Medical Systems, Horten, Norway).

### Statistical analysis

Data was tested for normality of distribution with the Kolmogorov- Smirnov test. Continuous variables are expressed as mean ± standard deviation or as median with an interquartile range (IQR with 25th to 75th percentiles). Categorical data are presented as absolute values and percentages. Comparisons between groups were performed with an independent t-test for continuous variables and a chi-squared test for categorical variables. Related echocardiographic parameters were compared using a Wilcoxon sign rank test. Pearson or Spearman correlation coefficients were used to determine correlations between VO_2_ peak and clinical, demographic and echocardiographic parameters.

Potential predictors for VO_2_ peak were entered in a multivariate linear regression model. A *P* value of less than 0.05 was considered statistically significant. All statistical analyses were carried out using the Statistical Package for the Social Sciences (SPSS version 28.0; SPSS Inc).

Our previous data has demonstrated that patients with severe aortic stenosis demonstrate a VO_2_ peak of 19.5 ml/min/kg (SD 5.9) [16]. For a difference of 25% between moderate cases and controls to be detected with an 80% power (alpha = 0.05, beta = 0.8) a sample size of 20 patients per group would be required. We allowed for an additional 5 patients per group to allow for the analysis of patients not demonstrating maximum effort ( RER < 1.1).

## Results

### Baseline demographics and echocardiographic data

65 patients with moderate AS were screened and 40 were excluded based on inability to cycle, co-existent valve disease and left ventricular impairment). 25 cases of moderate AS (Vmax 3.5 ± 0.2 m/s, MG 28 ± 5mmHg, AVA 1.2 ± 0.1cm^2^ ) were compared with 25 age and sex matched controls (age 63.2 ± 14.8 vs. 64.7 ± 8.1 years, *p* = 0.647, 64% male). Baseline demographics for all participants are displayed in Table 1. There were no significant differences in co-morbidities between the two groups and there was no known history of chronic lung disease in any of the participants. 11 patients with AS (44%) described NYHA class II symptoms of breathlessness. There were no reported symptoms of angina or syncope. The participants in the control group were asymptomatic at baseline.

**Table 1:** Baseline demographics for all participants

Parameter	Cases (*n* = 25)	Controls (*n* = 25)	*p* value
Age	63.2 ± 14.8	64.7 ± 8.1	0.647
Gender (male)	14 (64%)	14 (64%)	0.616
Hypertension	12 (48%)	9 (36%)	0.284
Diabetes	3 (12%)	9 (36%)	0.232
Hypercholesterolaemia	9 (36%)	8 (32%)	0.5
Coronary artery disease	8 (32%)	3 (12%)	0.085
Atrial fibrillation	2 (8%)	2 (8%)	0.695
Smoker	2 (8%)	0	0.245
Beta blocker therapy	5 (20%)	3 (12%)	0.702

The aetiology of the aortic stenosis was degenerative in 14 (56%), bicuspid in 10 (40%) and rheumatic in 1 (4%) patient (s) with AS. There was no concomitant valve pathology of more than mild severity. Patients with moderate AS had higher LVEDd (46 ± 7 vs. 41 ± 6, *p* = 0.004), LVMI (93 ± 29 vs. 75 ± 20, *p* = 0.014) and LAVI (38 ± 10 vs. 20 ± 4ml/m^2^) than controls. Baseline echocardiographic data are displayed in Table 2.

**Table 2:** Baseline echocardiographic data for all participants

Parameter	Cases (*n* = 25)	Controls (*n* = 25)	*p* value
Interventricular septum (mm)	11 ± 2	10 ± 2	0.204
Posterior wall (mm)	10 ± 2	10 ± 2	0.798
LVEDd (mm)	46 ± 7	41 ± 6	**0.004**
Relative wall thickness	0.45 ± 0.08	0.50 ± 0.13	0.099
LVMI	93 ± 29	75 ± 20	**0.014**
LVEDV (ml)	105 ± 17	95 ± 15	0.102
LVESV (ml)	47 ± 10	40 ± 10	0.203
LVEF (%)	61 ± 4	62 ± 4	0.631
Septal S’ (cm/s)	5.82 ± 2.00	5.71 ± 2.21	0.855
Lateral S’ (cm/s)	6.71 ± 2.67	6.43 ± 1.39	0.644
Average S’	6.27 ± 2.29	6.08 ± 1.60	0.727
GLS	15.7 ± 3.5	16.5 ± 3.2	0.565
RVIDd (mm)	35 ± 6	33 ± 5	0.471
TAPSE (mm)	22 ± 4	24 ± 3	0.495
E	0.78 ± 0.2	0.58 ± 0.1	0.292
E/A ratio	0.95 ± 0.38	0.93 ± 0.23	0.849
LAVI (ml/m^2^)	38 ± 10	20 ± 4	**0.002**
AVVmax	3.5 ± 0.2	1.8 ± 0.3	**< 0.001**
Mean gradient	28 ± 5	4 ± 1	**< 0.001**
AVA (cm^2^)	1.2 ± 0.1	2 ± 0.1	**< 0.001**
LVOT VTI	23.8 ± 4	24.7 ± 3	0.375
DVI	0.36 ± 0.14	0.6 ± 0.19	0.325
Indexed stroke volume	42 ± 9	45 ± 10	0.256

AVVmax; aortic valve peak velocity, AVA; aortic valve area, DVI; dimensionless index, LAVI; indexed left ventricular volume, LVEDd; left ventricular end diastolic diameter, LVMI; indexed left ventricular mass, LVEDV; left ventricular end diastolic volume, LVESV; left ventricular end systolic volume

*p* values in bold font indicate significant results

### Cardiopulmonary exercise echocardiography data

Cardiopulmonary exercise data are displayed in Table 3. There was a significant difference in peak heart rate and peak systolic blood pressure, between the cases and controls. Patients with moderate AS achieved a higher heart rate compared with controls (137 ± 20 vs. 126 ± 14 bpm, *p* = 0.004); however, the peak systolic blood pressure was higher in the control group (177 ± 23 vs. 188 ± 29mmHg, *p* = 0.027). There was no augmentation of systolic blood pressure on exercise in 2 patients with AS. 65% of participants reported a Borg score at the end of exercise of 0. The remaining 35% reported scores of 0.5, 1, 2 and 3.

**Table 3:** Cardiopulmonary exercise testing data

	Cases	Controls	*p* value
Height (cm)	171 ± 10	168 ± 10	0.277
Weight (kg)	77.5 ± 17	76.2 ± 13.2	0.774
Exercise time	10.7 ± 2.5	9.8 ± 1.8	0.175
Watts	116 ± 48	107 ± 25	0.405
Resting HR	73 ± 12	68 ± 10	0.103
Stress HR	137 ± 20	126 ± 14	**0.04**
Δ HR	64 ± 17	59 ± 16	0.294
SBP (rest)	133 ± 16	125 ± 15	0.107
SBP (stress)	177 ± 23	188 ± 29	**0.027**
Δ SBP	39 ± 20	64 ± 29	**< 0.001**
Peak VO_2_ (ml/kg/min) (all pts)	19.1 ± 4.6	18.8 ± 2.4	0.802
% predicted VO_2_ (all pts)	76 ± 15	84 ± 11	0.054
RER	1.16 ± 0.1	1.15 ± 0.7	0.140
Peak VO_2_ (ml/kg/min) in RER > 1.1	19.7 ± 4.4 (*n*=22)	19.0 ± 2.2 (*n*=22)	0.503
% predicted VO_2_ in RER > 1.1	77 ± 16	86 ± 31	0.208
VE/VCO_2_ slope	31.1 ± 8.7	26.9 ± 3.8	**0.030**
OUES	1703 ± 466	1755 ± 429	0.691
% predicted OUES	79 ± 15	89 ± 15	**0.013**
O_2_ pulse	11.0 ± 3.2	11.2 ± 2.5	0.832
% predicted O_2_ pulse	89 ± 18	99 ± 13	**0.019**
PETC0_2_	33 ± 4	38 ± 5	**0.001**
VO_2_ at AT (ml/kg/min)	14 ± 3.6	16.2 ± 3.3	**0.032**

*p* values in bold font indicate significant results

**Fig. 1:**
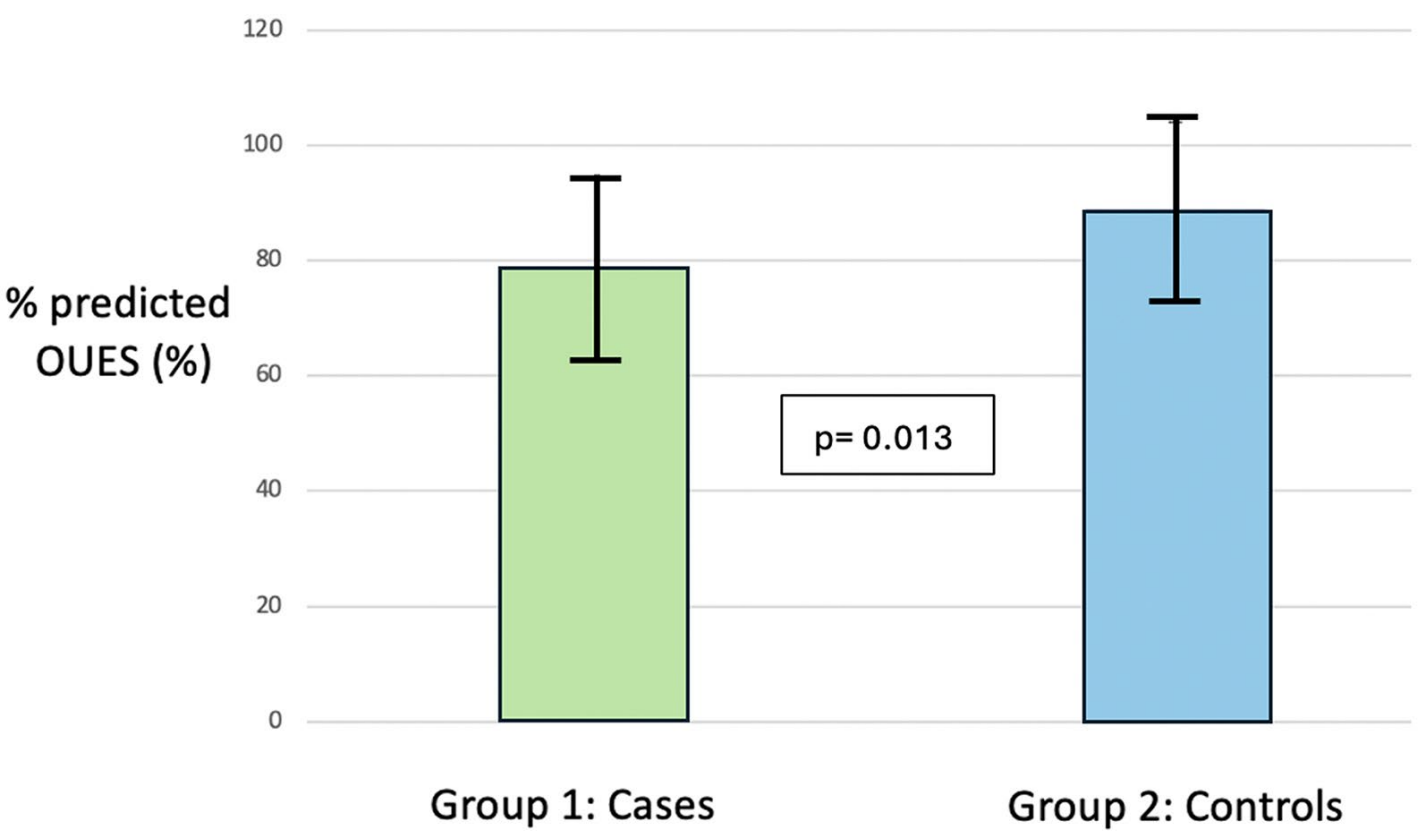
Comparison of % predicted OUES between cases and controls

44 participants (88%) performed a maximal exercise test and achieved an RER > 1.1. In these participants, the analysis for peak VO_2_ demonstrated no significant difference between patients with moderate AS, compared with controls (peak VO_2_ 19.7 ± 4.4 vs.19.0 ± 2.2 ml/kg/min, *p* = 0.503, 77 ± 16 vs. 86 ± 31% predicted, *p* = 0.208). For all 50 participants, there was no significant difference in absolute OUES values between cases and controls; however, the percentage predicted OUES was significantly higher in the control group (1703 ± 466 vs. 1755 ± 429, *p* = 0.691, 79 ± 15 vs. 89 ± 15% predicted, *p* = 0.013) (Fig. 1). The average VE/VCO_2_ slope was also higher in the AS patients compared with controls (31.1 ± 8.7 vs. 26.9 ± 3.8, *p* = 0.03). There were also significant differences in % predicted oxygen pulse, PETCO_2_ and VO_2_ at anaerobic threshold, between cases and controls. Out of the 44 participants that completed the exercise test to a good effort, 9 out 22 cases (41%) and 8 out of 22 controls (36%) exhibited a peak VO_2_ < 84% predicted. 13 out of 25 (52%) cases and 9 out of 25 controls had an OUES < 84% predicted. Two examples of 9 panel plots from participants with moderate AS and a control, are presented in Appendix 1.

**Fig. 2:**
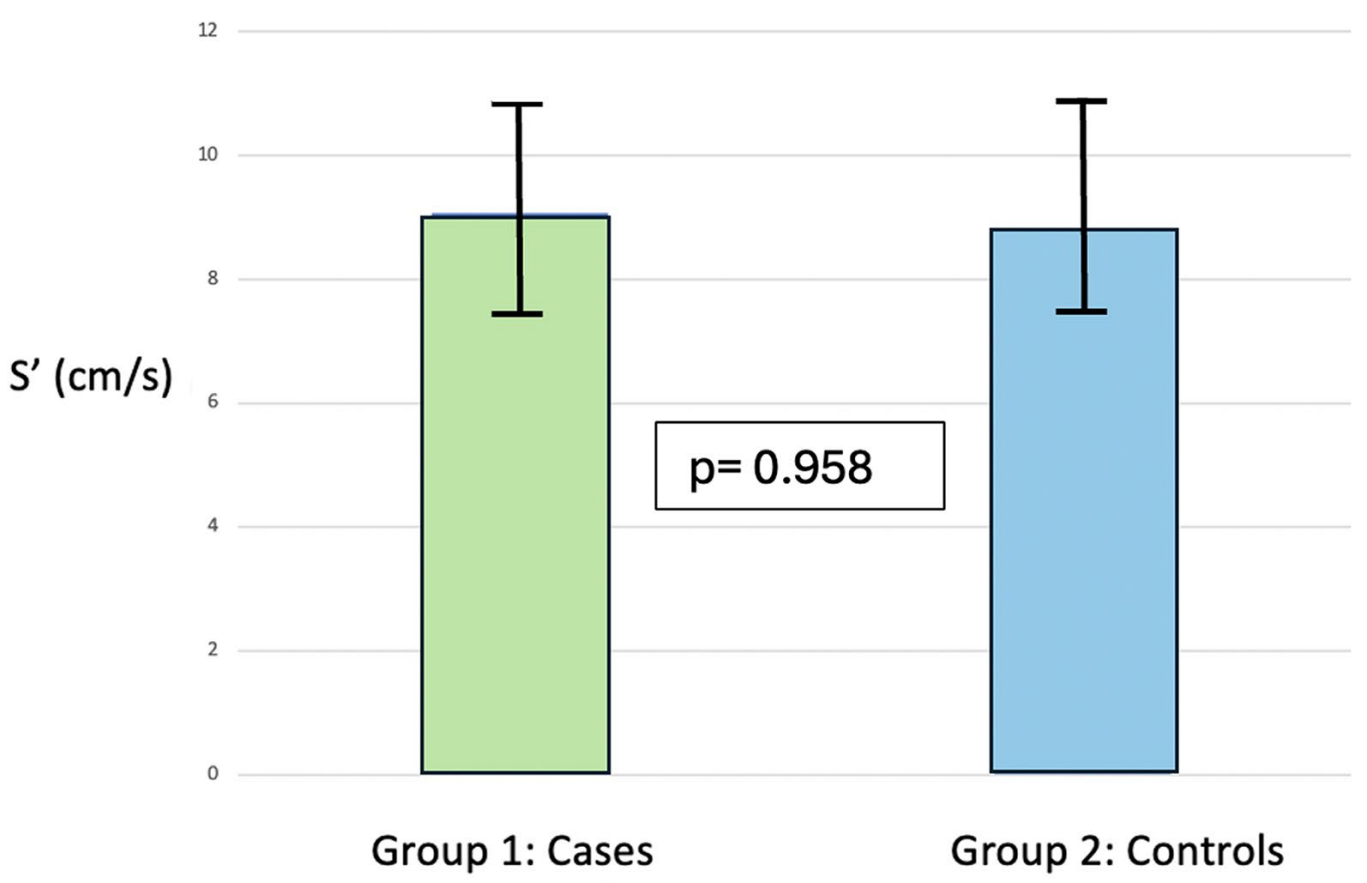
Comparison of peak systolic velocity (S’) between cases and controls

Exercise echocardiographic data are presented in Table 4. There were significant increases in Vmax and mean gradient in the patients with AS. The increase in LVEF was significant in all participants. Average S’ increased from 6.27 ± 2.29 to 9.07 ± 2.78 cm/s, *p* < 0.001, in patients with AS, and in controls 6.08 ± 1.60 to 8.83 ± 2.09 cm/s, *p* < 0.001) (Fig. 2). GLS increased from 15.7 ± 3.5 to 17.7 ± 2.9%, *p* = 0.041, in patients and 16.4 ± 3.2 to 19.2 ± 2.4% in controls, *p* = 0.001. There was no increase in pulmonary artery systolic pressure in the two groups.

**Table 4:** Exercise echocardiography data

	Cases	Controls	*p* value
AVVmax stress	4.1 ± 0.5	2.3 ± 0.4	**< 0.001**
Mean gradient stress	41 ± 14	7 ± 1	**< 0.001**
AVA stress	1.3 ± 0.3	2.3 ± 0.2	**< 0.001**
SVI			
LVEF (%)	69 ± 4	71 ± 3	0.636
Septal S’ stress (cm/s)	8.68 ± 2.84	8.33 ± 2.42	0.727
Lateral S’ stress (cm/s)	9.44 ± 2.99	9.40 ± 2.18	0.639
Average S’ stress (cm/s)	9.07 ± 2.78	8.83 ± 2.09	0.958
Δ S’	2.61 ± 2.06	2.61 ± 1.64	0.999
GLS	14.9 ± 3.9	19.2 ± 2.4	0.067
Δ GLS	3.4 ± 2.8	2.9 ± 2.2	0.580

*p* values in bold font indicate significant results

### CPET echocardiography parameters and symptoms at baseline

**Fig. 3:**
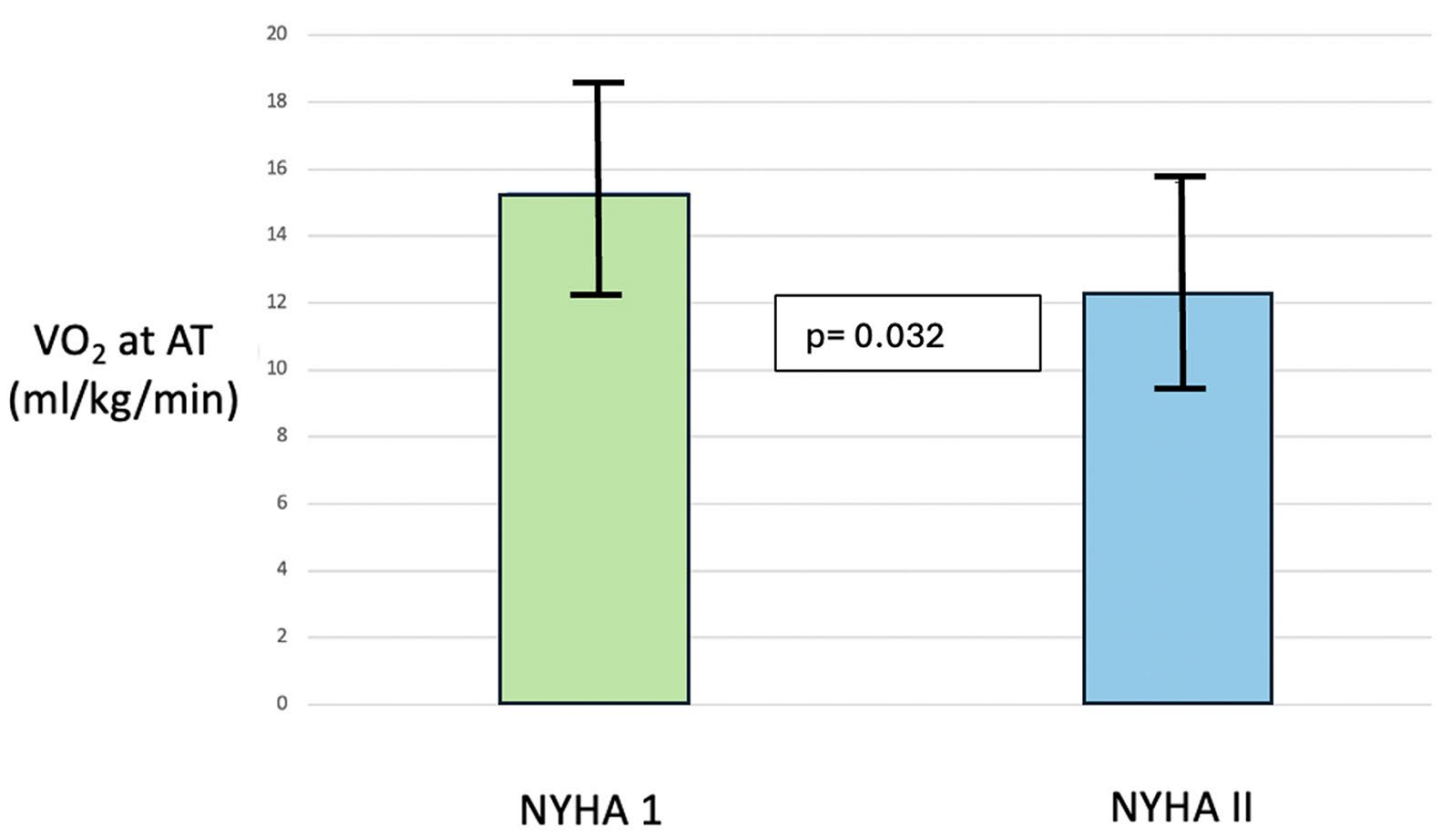
Comparison of VO_2_ at anaerobic threshold in patients with moderate AS and NYHA classes I and II

Table 5 displays the differences in CPET parameters between the patients with moderate AS with symptoms and those without symptoms, at baseline. There were no significant differences in peak VO_2,_ OUES, VE/VCO_2_ slope or oxygen pulse between patients with NYHA II symptoms compared with no NYHA I. However, a significantly lower VO_2_ at anaerobic threshold was observed in patients with NYHA II (NYHA I 15.3 ± 3.4 vs. NYHA II 12.3 ± 3.2 ml/kg/min, *p* = 0.020) (Fig. 3). Peak S’ was also lower in patients with NYHA II symptoms, compared to NYHA I (NYHA I 10.4 ± 2.7 vs. 7.4 ± 1.8 cm/s, *p* = 0.005).

**Table 5:** Relationship between CPET and echocardiographic variables with NYHA class

Parameter (AS patients only)	NYHA I *n* = 14	NYHA II *n*= 11	*p* value
Peak VO_2_ (in patients with RER > 1.1)	20.2± 4.7	19.2 ± 3.8	0.585
% predicted VO_2_ in patients with RER > 1.1	75 ± 14	79 ± 17	0.452
VE/VCO_2_ slope	29.9 ± 10	32.6 ± 6.8	0.442
OUES	1808 ± 490	1567 ± 417	0.227
% predicted OUES	80 ± 15	76 ± 15	0.542
Oxygen pulse	10.9 ± 3.1	11 ± 3.6	0.936
% predicted oxygen pulse	89 ± 15	88 ± 21	0.956
PETCO_2_	34.1 ± 4.2	33.3 ± 4.8	0.654
VO_2_ at AT	15.3 ± 3.4	12.3 ± 3.2	**0.020**
AVVmax	3.5 ± 0.3	3.4 ± 0.2	0.201
Mean gradient	28 ± 5	28 ± 4	0.933
AVA	1.2 ± 0.2	1.1 ± 0.2	0.300
Average S’ rest	6.9 ± 2.5	5.5 ± 1.8	0.126
Average S’ stress	10.4 ± 2.7	7.4 ± 1.8	**0.005**
Δ S’	3.3 ± 1.7	1.7 ± 2.2	0.056
GLS rest	15.8 ± 3.4	14.3 ± 3.1	0.510
GLS stress	16.9 ± 2.2	17.1 ± 3.4	0.901
Δ GLS	4.2 ± 2.5	2.8 ± 3.7	0.457

*p* values in bold font indicate significant results

## Discussion

The results from this cardiopulmonary stress echo study demonstrate that:

- a large proportion of patients with moderate AS and preserved LVEF (44%) report symptoms.
- peak VO_2_ was < 84% predicted in 41% of patients and OUES was < 84% predicted in 52% of patients with moderate AS.
- patients with moderate AS demonstrate objectively reduced exercise intolerance compared with age and sex matched controls (% predicted OUES, % predicted O_2_ pulse, VO_2_ at anaerobic threshold).
- VE/VCO_2_ is higher in patients with moderate AS compared with controls.
- VO_2_ at anaerobic threshold is lower in patients with moderate AS with NYHA II symptoms at baseline, compared with patients in NYHA I.

### Outcomes in moderate AS

There is increasing evidence that less severe forms of aortic stenosis are associated with premature mortality, in some cases approaching that of severe AS [8]. Otto et al. carried out a natural history studies of 123 patients with asymptomatic AS and divided them into 3 groups, according to baseline Vmax < 3, 3–4 and > 4 m/s. They showed that the likelihood of remaining alive without valve replacement at 2 years was only 21 ± 18% (Vmax > 4 m/s), 66 ± 13% (Vmax 3–4 m/s) and 84 ± 16 (Vmax < 3 m/s). Event free survival of patients with a Vmax 3–4 m/s decreased drastically after 2–3 years [6]. Although it has been shown that patients with moderate AS and reduced left ventricular ejection fraction have an increased mortality [17–19], patients with preserved LVEF are also at increased risk of cardiovascular events [20].

Early AVR for moderate AS may result in lower all-cause mortality. A recently published systematic review and meta-analysis of 7 observational studies (4827 patients) comparing early AVR with conservative management in patients with moderate AS demonstrated that occurrence of AVR and early AVR was associated with a 45% decreased risk of all-cause mortality (HR = 0.55 [0.42–0.68], *I*^2^ = 51.5%, *P* < 0.001). Three studies included patients with LVEF < 50%, one study included patients with LVEF > 50% and three studies included a range of LVEF [21]. A further meta-analysis from this year showed that occurrence of AVR and early AVR for moderate AS were associated with significantly lower all-cause mortality while LVEF < 50% and symptomatic status were associated with an increased all-cause mortality [22].

### Symptoms and exercise capacity in moderate AS

The three main symptoms of AS are dyspnoea (most common), angina and syncope. The compensatory mechanisms in AS start at an early stage of the disease process, leading to diastolic dysfunction and symptoms, even when LVEF is preserved [23].

A study by Stassen et al. in 1961 patients with moderate AS demonstrated that patients with even mild symptoms (NYHA II) have an increased risk of adverse events, even in the presence of a normal LVEF [24].

Exercise testing has been shown to be safe and feasible in patients with AS and 23% of patients with moderate AS exhibited symptoms on treadmill testing [25]. The data regarding the use of exercise echocardiography in moderate aortic stenosis is limited. An exercise induced increase in aortic mean gradient > 20mmHg was associated with a faster progression of AS [26], and patients with a resting mean gradient of > 35mmHg and exercise induced increase in mean gradient > 20mmHg were at a higher risk of death or AVR during a mean follow up of 20 months [27].

We have demonstrated that in patients with mild symptoms of breathlessness (NYHA II), VO_2_ at anaerobic threshold and peak S’ are significantly lower, compared with patients in NYHA I. These findings may suggest that the aortic valve is the driver of symptoms and that closer surveillance of aortic stenosis or even intervention is warranted even when the patient begins to develop mild symptoms.

### Cardiopulmonary exercise testing

VO_2_ peak is frequently used as the most reliable measure of overall exercise capacity and is strongly influenced by the patient’s motivation and the testers subjective choice of endpoint [28]. There are other measures of exercise performance that can be interpreted in the context of a submaximal exercise test. The anaerobic or ventilatory threshold is a marker that identifies the onset of significant muscular anaerobic metabolism; however, it can be difficult to obtain in and can be subject to interobserver variability [29].

The oxygen uptake efficiency slope (OUES) proposes a submaximal analysis of the values obtained on a cardiopulmonary test. It is based on the curvilinear relationship between the minute ventilation and the oxygen uptake throughout an incremental CPET. Baba introduced a log transformation resulting in a linear relationship between the minute ventilation and the oxygen uptake during the entire and especially the last part of the CPET. The theoretical linearity of the OUES implies that it does not require a maximal test. Interobserver variability is minimised as the OUES is determined mathematically, and correlates highly with peak VO_2_ (*r* = 0.941, *p* < 0.001) [30]. It has prognostic value in predicting major cardiac events and mortality in patients with coronary artery disease [31] and heart failure [32], more so than peak VO_2_.

To our knowledge, there have been no published studies exploring the value of OUES in patients with aortic stenosis. Our findings demonstrated a significantly lower % predicted OUES in patients with moderate AS, compared with controls.

Ventilatory efficiency (VE/VCO_2_) during exercise is defined as the ratio of minute ventilation (VE) and carbon dioxide output (VCO_2_) and reflects matching of alveolar ventilation and pulmonary perfusion [33]. It is calculated using a regression function and as the slope from rest to anaerobic threshold is linear, a more accurate assessment can be made in patients with a submaximal exercise test. The VE/VCO_2_ slope provides independent prognostic information in addition to peak VO_2_ and is closely related to symptoms [34].

Levy et al., studied 43 patients with asymptomatic AS that underwent CPET and found 74% patients achieved an RER > 1.15 and that a VE/VCO_2_ slope > 34 was associated with an abnormal exercise test (defined by symptoms, fall in blood pressure or ventricular arrhythmias) or development of AS attributable symptoms at follow up [35]. The prognostic of VE/VCO_2_ slope was subsequently studied in AS and found to be the only independent predictor of decompensated heart failure, syncope and mortality [36]. Both of these studies included patients with severe AS, as defined by the current guidelines [1–2].

Oxygen pulse is the VO_2_/HR ratio and reflects the amount of oxygen consumed per heartbeat and is a correlate of stroke volume reserve. A flattening or downward displacement of oxygen pulse kinetics during incremental exercise may reflect cardiac limitation [37]. Levy’s study found that oxygen pulse did not predict an abnormal exercise test, probably because a large number of patients were on beta blocker therapy [35]. A decreased oxygen pulse has been found to have prognostic value in studies of moderate to severe AS [38–39]. We found that % predicted oxygen pulse was lower in cases compared with controls. However, the peak heart rate was significantly higher in patients compared with controls, which may have led to a lower O_2_ pulse.

### Echocardiography in moderate AS

Moderate AS is associated with adverse LV remodeling and the degree of remodelling has been shown to approach that of severe AS. Left ventricular hypertrophy, myocardial fibrosis, hypertrophy, diastolic dysfunction, left atrial dilatation and pulmonary hypertension are all independently associated with significant morbidity and mortality, in moderate AS [40–41].

LV global longitudinal strain is a more robust marker of left ventricular function than LVEF.

Several studies have evaluated the prognostic value of GLS in patients with moderate AS. A GLS cut off value of 15.2% associated with higher mortality rates in patients with moderate AS and preserved LVEF [42]. A further study by Stassen demonstrated that in patients with moderate AS and LVEF ≥ 50%, a GLS cut off value of < 16% was associated with a lower survival, compared with patients with GLS ≥ 16% [43].

### Limitations

The main limitation of this study is the small sample size. This was a single centre study and therefore the results cannot be generalised to the wider population. However, to our knowledge, this is the first study to explore the use of cardiopulmonary stress echocardiography in moderate AS, prospectively, and with a non-AS control group.

## Conclusions

A large proportion of patients with moderate AS are symptomatic, and display objective evidence of exercise limitation, on cardiopulmonary exercise testing. By matching with controls, we have demonstrated that the difference in % predicted OUES, % predicted oxygen pulse and VO_2_ at anaerobic threshold in patients with moderate AS, is attributed to the AS and not other co-morbidities or characteristics. These findings emphasise the benefit of objective testing with CPET and the value of submaximal parameters in those patients unable to complete a maximal test.

Moderate AS is associated with significant cardiovascular morbidity and mortality in large observational studies. This has important clinical implications; whether the current indications for AVR should expand in cases of less severe AS remains unknown. A risk stratification model incorporating exercise echo and CPET could improve identification of patients at an increased risk of adverse events that may benefit from more intense follow up. The results of ongoing randomised controlled trials investigation the benefit of AVR or TAVI in patients with moderate AS are awaited.

## Electronic supplementary material

Below is the link to the electronic supplementary material.

Supplementary Material 1

## Data Availability

No datasets were generated or analysed during the current study.
