# Supplementary material for: Exercise capacity in moderate aortic stenosis: a cardiopulmonary stress echocardiography study
Source: Echo Res Pract. 2025 Mar 5;12:6. doi: 10.1186/s44156-025-00070-7 (PMC11881479; doi:10.1186/s44156-025-00070-7)
Supplement: Supplementary file 1 — Supplementary Material 1 [file 44156_2025_70_MOESM1_ESM.docx]

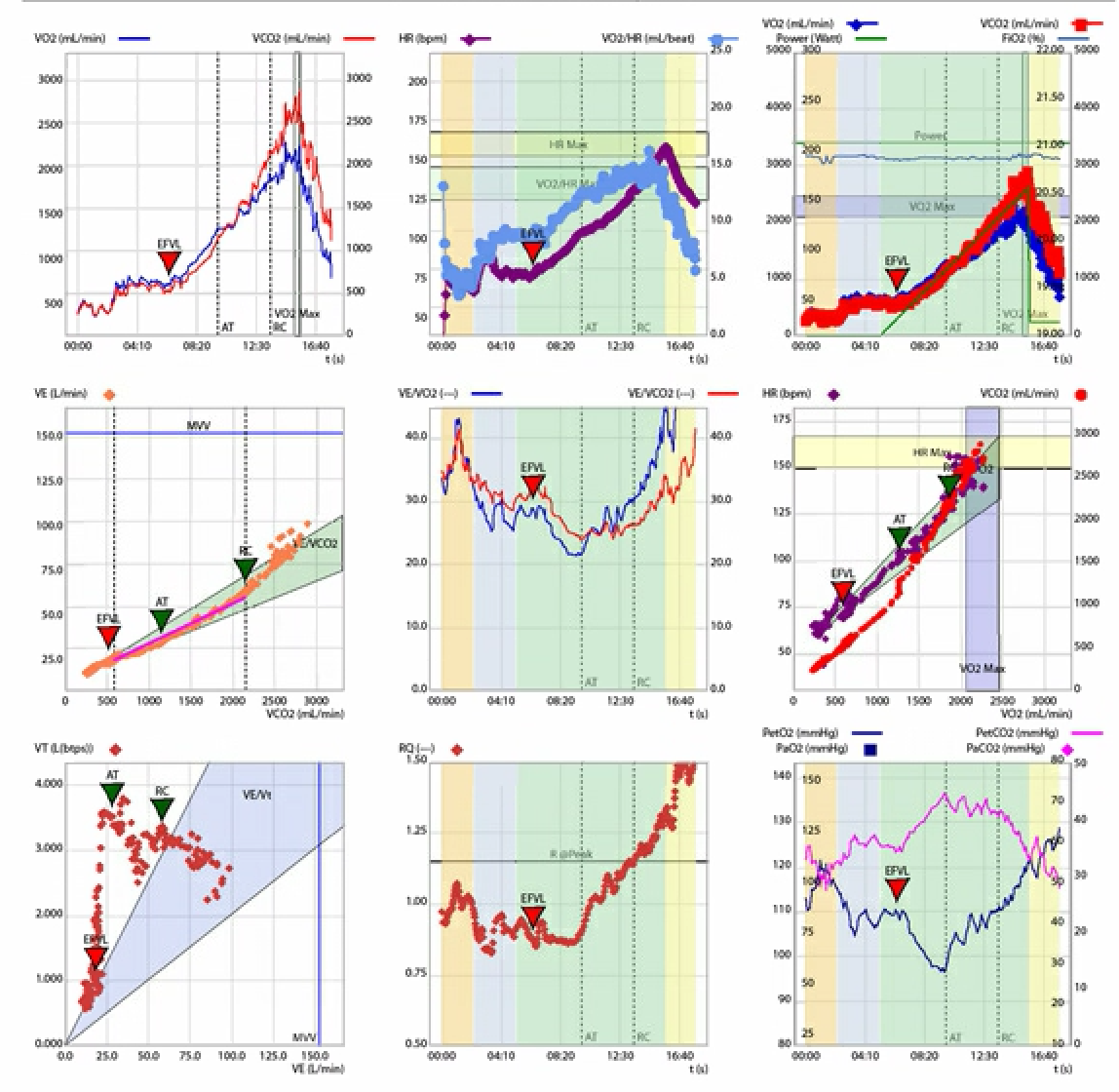


Traditional 9 panel plot of a control participant exhibiting good exercise tolerance.


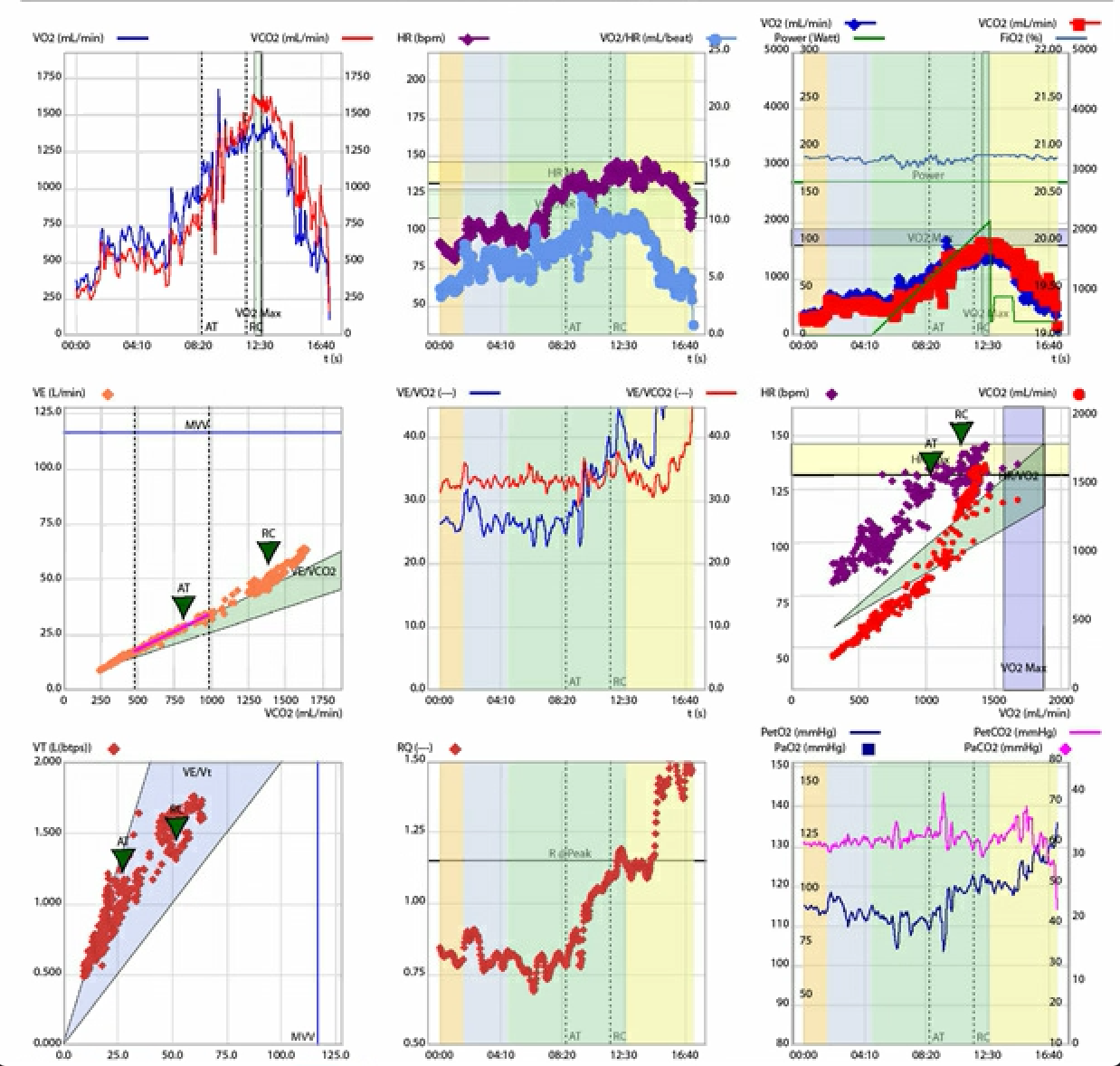


Traditional 9 panel plot of a patient with moderate aortic stenosis exhibiting reduced exercise tolerance
